# Understanding Stakeholder Views Regarding the Design of an Intervention Trial to Reduce Anticholinergic Burden: A Qualitative Study

**DOI:** 10.3389/fphar.2021.608208

**Published:** 2021-11-12

**Authors:** Yvonne Cunningham, Karen Wood, Carrie Stewart, Athagran Nakham, Rumana Newlands, Katie I. Gallacher, Terence J. Quinn, Graham Ellis, Richard Lowrie, Phyo Kyaw Myint, Christine Bond, Frances S. Mair

**Affiliations:** ^1^ Institute of Health and Wellbeing, College of Medical, Veterinary and Life Sciences, University of Glasgow, Glasgow, United Kingdom; ^2^ School of Medicine, Medical Sciences and Nutrition, University of Aberdeen, Aberdeen, United Kingdom; ^3^ Health Services Research Unit, University of Aberdeen, Aberdeen, United Kingdom; ^4^ Academic Geriatric Medicine, New Lister Building, Glasgow Royal Infirmary, Glasgow, United Kingdom; ^5^ NHS Lanarkshire, Bothwell, United Kingdom; ^6^ Pharmacy Services, NHS Greater Glasgow and Clyde, Glasgow, United Kingdom; ^7^ The School of Medicine, Medical Sciences and Nutrition, University of Aberdeen, Aberdeen, United Kingdom

**Keywords:** anticholinergics, deprescribing, polypharmacy, interviews, focus groups, qualitative research

## Abstract

**Background:** Anticholinergic burden (ACB), is defined as the cumulative effect of anticholinergic medication which are widely prescribed to older adults despite increasing ACB being associated with adverse effects such as: falls, dementia and increased mortality. This research explores the views of health care professionals (HCPs) and patients on a planned trial to reduce ACB by stopping or switching anticholinergic medications. The objectives were to explore the views of key stakeholders (patients, the public, and HCPs) regarding the potential acceptability, design and conduct of an ACB reduction trial.

**Materials and Methods:** We conducted qualitative interviews and focus groups with 25 HCPs involved in prescribing medication with anticholinergic properties and with 22 members of the public and patients who were prescribed with the medication. Topic guides for the interviews and focus groups explored aspects of feasibility including: 1) views of a trial of de-prescribing/medication switching; 2) how to best communicate information about such a trial; 3) views on who would be best placed and preferred to undertake such medication changes, e.g., pharmacists or General Practitioners (GPs)? 4) perceived barriers and facilitators to trial participation and the smooth conduct of such a trial; 5) HCP views on the future implementability of this approach to reducing ACB and 6) patients’ willingness to be contacted for participation in a future trial. Qualitative data analysis was underpinned by Normalization Process Theory.

**Results:** The public, patients and HCPs were supportive of an ACB reduction trial. There was consensus among the different groups that key points to consider with such a trial included: 1) ensuring patient engagement throughout to enable concerns/potential pitfalls to be addressed from the beginning; 2) ensuring clear communication to minimise potential misconceptions about the reasons for ACB reduction; and 3) provision of access to a point of contact for patients throughout the life of a trial to address concerns; The HCPs in particular suggested two more key points: 4) minimise the workload implications of any trial; and 5) pharmacists may be best placed to carry out ACB reviews, though overall responsibility for patient medication should remain with GPs.

**Conclusion:** Patients, the public and HCPs are supportive of trials to reduce ACB. Good communication and patient engagement during design and delivery of a trial are essential as well as safety netting and minimising workload.

## Introduction

### Background

Medications with anticholinergic properties are commonly prescribed for a broad range of health conditions and are a group of drugs that are particularly associated with adverse events (Hanlon et al., 2020). Medications with anticholinergic effects are used to treat a wide range of symptoms including those affecting the urinary system and bladder, respiratory system, mental health and certain diseases such as Parkinson’s disease (Chew et al., 2008). Such medications are also frequently prescribed for many chronic conditions including high blood pressure, heart disease, hay fever and chronic lung disease. Many different classes of medications including analgesic and anti-inflammatory agents, anti-depressants, antidiabetic agents, anti-infectives, antipsychotics, anxiolytics and sedative hypnotics, cardiovascular medications, gastrointestinal and bowel agents, anticonvulsants and urinary incontinence agents have anticholinergic properties. Supplementary File two provides a list of many commonly prescribed medications with anticholinergic properties. They continue to be commonly prescribed in 20–30% of older people despite their known association with negative health-related outcomes (Singh, Lake and Furberg, 2008; Fox et al., 2014; Myint et al., 2015; Richardson et al., 2015). The risk varies greatly between medications and accumulates with polypharmacy to create a measurable level of anticholinergic burden (ACB). Increasing ACB is associated with a range of adverse effects ranging from falls through to cardiovascular events, dementia and death (Tune, 2001; Hanlon et al., 2020). Research is urgently needed to aid clinicians in measuring and reducing ACB in older people if we are to reduce the associated adverse health outcomes. Policy documents have highlighted risks of anticholinergic medications (Scottish Government, 2018; Scottish Government, 2021) and promote deprescribing efforts. However, the best approach to deprescribing and the precise benefits or risks of deprescribing interventions remain uncertain. Hence there is an urgent need for clinical trial evidence, such as that from an ACB deprescribing/medication switching trial, to inform deprescribing efforts in clinical practice.

There are several scales that measure cumulative ACB (Neal et al., 2017; Graves-Morris et al., 2020), however, a key uncertainty is the question of how best to reduce ACB burden at both population and individual patient level and importantly, whether interventions to reduce ACB improve health. Any intervention that aims to tackle prescribing practice in older adults requires a multidisciplinary approach and any ACB reduction trial will fall into the category of a complex intervention as it will involve a number of different interacting components, including influencing the behaviour and work of health care professionals (HCPs) and patients (Craig et al., 2008). A key aspect of intervention development is involving key stakeholders, in this case, patients, the public and HCPs, from the outset (O’Cathain et al., 2019). The literature on barriers and facilitators to reducing ACB is scant (Stewart et al., 2019) and to inform design of a proposed ACB reduction trial it will be important to explore the views of such key stakeholders regarding the potential acceptability and design of such an intervention.

The objectives of this research were to explore the views of such key stakeholders (i.e. patients, the public and HCPs) regarding the potential acceptability, design and conduct of a planned trial to reduce ACB by stopping or switching anticholinergic medications. This qualitative research is an essential step towards development of a definitive trial to inform prescribing practice and policy.

### Methods

#### Design

We conducted a qualitative study involving semi-structured interviews and focus groups with healthcare professionals working within two health boards in Scotland (NHS Greater Glasgow & Clyde and NHS Grampian), and members of the public and patients recruited from Glasgow, Edinburgh and the West of Scotland, UK, between August 2019 and January 2020.

#### Participants

Eligible patients were those aged 65 years and over, who have been prescribed anticholinergic medications and were able to provide informed consent. Specifically, patients were eligible if they took (or had taken in the past) two or more medications from the list of medications with anticholinergic properties provided in Supplementary File S2.

Eligible HCPs had to be employed as clinicians/doctors or pharmacists within primary or secondary care within NHS Greater Glasgow and Clyde or NHS Grampian, and whose role involved providing care to patients aged 65 years and over. A full list of the roles of the HCPs interviewed is provided in Table 1.

**TABLE 1:** Breakdown of HCP roles of participants.

Healthcare professional	Greater Glasgow & Clyde^a^ (n)	Grampian^b^ (n)
GP	6	2
Trainee GP	2	0
Geriatrician	4	4
Pharmacist	4	3

a NHS Greater Glasgow and Clyde is one of 14 regional NHS Boards in Scotland. It is the largest health board in the UK.

b NHS Grampian provides NHS services for the half-million people who live in Grampian and are overseen by one single NHS board.

Ethical approval was obtained from both study centres before starting public and patient recruitment and participants signed a written informed consent before participation.

#### Recruitment

Members of the public were recruited from patient and carers’ support groups, including the Alliance Scotland, the Glasgow Stroke Group, and NKS (Nari Kallyann Shangho). Potential participants from all three groups received an e-mail containing a flyer advertising the opportunity and listing the eligibility criteria, please see the flyer in Supplementary File S2.

The Health and Social Care Alliance Scotland (the Alliance) is Scotland’s national third sector intermediary for a range of health and social care organisations. Their membership encompasses over 3,000 national and local third sector organisations, associates in the statutory and private sectors, disabled people, people living with long-term conditions and unpaid carers. The Alliance advertised through their mailing list of members to recruit people who would be interested in participating in focus groups about reducing ACB.

The Glasgow Stroke Research Group is a group of patients who have experienced a stroke or who care for somebody who has had a stroke. The group members are all interested in being involved in PPI (Patient and Public Involvement in research) and many of the medications prescribed for patients who have had a stroke have anticholinergic properties. The Glasgow Stroke Research Group is part of the NHS Research Scotland Stroke Research Network. The group advertised through their mailing list of members for anyone who would be interested in participating in a focus group about reducing ACB.

NKS (Nari Kallyann Shangho) is a health advocacy group for South Asian women and their families. NKS provide many services including a carer’s forum, education and training including English to Speakers of Other Languages (ESOL) and informal computer classes, volunteering opportunities and work placements, family support, home visits, and they also run a nursery and after-school club. NKS advertised to their service users for anyone interested in taking part in a focus group or interview about reducing ACB.

Patient interviewees were also recruited from primary care practices with the assistance of the NHS Research Scotland (NRS) Primary Care Network. Eight men and fourteen women, aged between 65 and 83, participated. Of these, twelve participated in one of three focus groups, and ten participated in one-to-one semi-structured qualitative interviews.

Recruitment of HCPs involved multiple strategies and purposive sampling to ensure representation of HCPs from differing geographical areas and health care settings (i.e., primary and secondary care). General Practitioners (GPs), geriatricians and pharmacists were recruited because they all provide support to patients with chronic illness and are involved in prescribing and medication management. All three groups of professionals are also involved in de-prescribing efforts.

Potential participants were identified using the contacts and networks of the Glasgow and Aberdeen research teams and the NRS Primary Care Network. In total, twenty five participants were included. Please see Table 1 for more information on participant characteristics. Of these, fourteen participated in a semi-structured qualitative interview and eleven participated in one of three focus groups.

#### Procedures

We worked with support from contacts at the NRS Stroke Group, NKS and the Alliance. We emailed these contacts an invitation pack containing a printable flyer to advertise the upcoming focus groups, a Participant Information Sheet and a letter of invitation. Our contacts passed this information onto potential participants and when they had gathered enough recruits organised times and dates for the focus groups and interviews to take place with us.

For patients recruited through primary care, patients were sent an invitation pack containing a letter of invitation, Participant Information Sheet, expression of interest form and a stamped addressed envelope. Those interested in participating in the study were asked to return their expression of interest forms using the stamped addressed envelopes provided. Potential participants were then contacted by YC who provided further information on the study and answered any questions they might have. If the potential participant decided they would like to participate they were asked about their medical conditions and medications and agreed a convenient date, time and location for an interview or focus group. The researcher (YC) made three attempts to contact potential participants (at varying times and days), if no response was received, another potential participant was selected.

Interviews were conducted until data saturation was reached (i.e., no new themes within the data emerged) (Francis et al., 2010). Before beginning each interview or focus group signed informed consent was obtained. Interviews and focus groups were recorded and transcribed verbatim for analysis.

HCPs who expressed an interest in participating were emailed an invitation pack containing a letter of invitation, Participant Information Sheet and expression of interest form.

Those expressing interest were emailed with details of the date and time of the focus group and asked to confirm acceptance. If no acceptance was received, a reminder email and/or phone call was made. At the time of the interview or focus group the researcher (YC) reconfirmed consent verbally with the participants. Interviews and focus groups were recorded and transcribed verbatim providing the data for qualitative analysis.

Each focus group began by welcoming participants and making introductions. This was followed by a short presentation from the research team to explain the purpose of the focus group in more detail and to give a brief overview of ACB. Participants were then given the opportunity to ask questions before being invited to discuss their views about ACB and de-prescribing or switching medications to reduce or lessen ACB. The Topic Guide (which is included in Supplementary File S3) was used to structure the discussion.

#### Development of Interview Schedule

In our interviews and focus groups, we used a Topic Guide which explored several aspects of feasibility including: 1) views of a trial of de-prescribing/medication switching; 2) how to best communicate information about such a trial; 3) views on who would be best placed and preferred to undertake such medication changes; 4) perceived barriers and facilitators to trial participation and the smooth conduct of such a trial; 5) HCP views on the feasibility of this approach to ACB reduction and 6) willingness to be contacted for participation in a future trial of this kind. The Topic Guide is available in Supplementary File S3.

#### Data Analysis

Normalization Process Theory (NPT) was used to underpin the analysis of interview and focus group transcripts. NPT explains how the work of enacting an ensemble of tasks or practices is accomplished through the operation of four mechanisms: “coherence” (sense-making work); “cognitive participation” (relationship work); “collective action” (enacting work); and “reflexive monitoring” (appraisal work) (May and Finch, 2009; May et al., 2009; Murray et al., 2010). We conceptualised the reduction of ACB as a complex process comprising these four types of mechanisms. Transcripts were imported into the software package NVivo, version 11 (QSR International, Melbourne, VIC, Australia). Following familiarisation with the transcripts by YC, FM and CS, data were analysed using thematic analysis; a method for searching, identifying and analysing patterns of meaning or themes, in a dataset (Daly, Kellehear and Gliksman, 1997; Braun and Clark, 2006). Thematic analysis has six phases: familiarisation with data, generating initial codes, searching for themes, reviewing themes, defining and naming themes, and producing the report (Braun and Clark, 2006).

In our data analysis we followed a hybrid approach of qualitative methods of thematic analysis, drawing on both inductive (i.e., data-driven) and deductive (i.e., based on pre-conceived ideas) approaches. This was an iterative and reflexive process with the data collection and analysis being conducted concurrently. Indeed, we chose thematic analysis among other qualitative methods for its flexibility, while we position our study within the social constructionist epistemological tradition, according to which patterns of meaning and experience are socially produced and reproduced (Braun and Clarke, 2006).

An initial thematic coding frame was developed. The themes were based on NPT as per the topic guide. YC and CS coded all the transcripts to produce a summary report and FM reviewed the summary and discussed it with YC to check consistency with the data.

### Results

Our findings are presented under 6 key themes: 1) Sense-making (patient and professional views of a trial of de-prescribing/medication switching); 2) Relationship Work (patient and professional views on how to best communicate information about such a trial); 3) Enacting Work (patient and professional views regarding who should undertake such medication changes, for example, pharmacists or GPs?); 4) Perceived barriers and facilitators to trial participation and the smooth conduct of an ACB reduction trial; 5) Appraisal Work (HCP and patient views on the future execution of this approach to ACB reduction; and 6) Appraisal Work (Reflexive monitoring): Public and patients’ willingness to be contacted for participation in a future trial of this kind and general views about a trial. These themes are described, and illustrative quotations provided in the following section.

1) Sensemaking (coherence): patient and professional views of a trial of de-prescribing/medication switching.

For patients, the sense-making or “coherence” work involved learning about their conditions and medications, they described getting information from GPs, pharmacists and HCPs, as well as “Dr Google” and friends and family members and of wanting a better understanding of why they were taking different medications.

And when you, the internet is the worst thing ever, you know you can go onto it and you look up all these things and you can fit yourself into them (overtalk), well you can fit your symptoms into them, right, and you can say: “Oh I’ve got that” you know, “Oh I’ve got that as well” right and you know you think you just had the cold right, oh no it’s you know, you know it’s some dengue fever or something, or other you know the Black Death has come back.

AFG2M11, patient participant at focus group

Some patients read the information leaflet which came with their medications thoroughly, others discarded it, preferring not to learn about potential side-effects as they felt that knowing might make them hesitant to take the medication. Patients who were interviewed generally held positive views about the possibility of running a trial of de-prescribing/medication switching.

For HCPs, making sense of ACB first involved increasing their awareness of the issues (knowledge and experience in dealing with ACB varied depending on their roles). GPs described the challenge of adding yet another factor that they needed to bear in mind when prescribing as well as the problem of any ACB reduction trial adding to an already heavy workload.

I think I pretty much covered that already in that it’s a very good thing to do. I guess we would need to think about the time factors, already there’s GP pressures, we’ve got 10-min appointments, how would we do this extra task? We’re constantly being asked from lots of different professionals to do different tasks. We’ve got to have an (inaudible) care plan, got to do the anticholinergic burden, we’ve got to do a polypharmacy review, multi morbidity review, we’ve got to do an annual reviews for every single condition and that’s not even addressing any of the patient’s agenda. It’s really difficult if you put all of this on the doctor’s agenda because in 10 minutes we barely have time to get through what the patients are worried about. I think, yeah, it’s challenging.

Participant 9, GP in Aberdeen

HCPs believed that de-prescribing/switching would be good for patients in the long run, and that findings from a trial could go towards building the capacity of the health service to do this.

2) Relationship work (cognitive participation): patient and professional views on how to best communicate information about such a trial.

The work done to build and maintain the relationships between patients and HCPs was considered vital by both groups. Patients felt this could be facilitated by HCPs having more time to discuss and explain medications and side-effects with their patients.

“How do you talk to somebody in 10 min?” you go in and you’re maybe wanting to tell them something right, but you know, you end up coming out not having told them, but that’s the thing, if they had a wee bit more time but restrictions, you know if they had maybe 20-min appointments right, but the numbers of GPs aren’t sufficient for that.

AFG2M11, patient participant at focus group

Patients who were interviewed and who participated in focus groups made a range of suggestions for how recruitment for a future trial could be carried out, including letters from GPs, face-to-face approaches at clinics, working together with voluntary sector patient representative organisations and using social media.

Well I actually think the Facebook messages are good to be honest, you know I see quite a lot of things on their trials, and I’ve gone for quite a few of them, I’ve not been suitable for them because you know, but you see things like: “Do you have diabetes and high blood pressure?” for instance, “Would you be prepared … ?” and that’s, I actually think those are, I don’t, I know that interests me, I don’t know how many other people it interests, but I’ve found that quite a good way of contacting people. Yeah, and probably the GP as well, if the GP said to me: “Now, we’ve got a trial for exactly what you’re on, would you be interested?” I mean I would say immediately yes, I don’t know about other people.

AF15, patient interviewee

3) Enacting work (collective action) - Patient and Professional views regarding who should undertake such medication changes, for example, pharmacists or GPs.

It was clear that both patients and HCPs appreciated that any intervention would need them to act differently. HCPs suggested there would be a greater need for them to explain and discuss the new way of working which would take time. Some patients felt they should take a more active role in their healthcare, but at the same time did not want to take up too much of their GP’s time.

I think when you’re, when you’re reviewing someone’s medications, depending on the person, there’s a lot of work and time to be spent explaining: “Why are you on this?” “What’s this doing for you?” “What are your symptoms?” “This is what we’re trying to do,” I think it’s just the main burden, just having time to do that, and to do that, and to do that successfully with, with patients on a kind of one-to-one because everyone’s going to be on a different kind of cocktail of things.

TFGFP18, GP in Glasgow

HCPs mentioned that any intervention would involve adapting to changes in roles and responsibilities, as for example, pharmacists may take over prescribing responsibilities from GPs. Most patients who were interviewed and who participated in focus groups felt that the GP should be the person to advise and carry out any medication changes, however, patients who attended practices which included a primary care pharmacist (who can currently carry out reviews of medication in Scotland) said they were comfortable with a pharmacist undertaking medication changes.

I would appreciate either a GP review, or a pharmacy review, and I’m not knocking the nurse right, but I don’t think the nurse has enough in-depth understanding of medication, you know because, pharmacy it’s about 5 years right talking about interaction of drugs and that, medicine it’s 5 years and there’s a wee bit pharmacology in it as well, whereas nursing there’s not really any pharmacology in their training right, so that’s just my personal view you know.

AFG2M11, participant at focus group

The HCPs interviewed differed with each other in their views about who should undertake medication changes. Pharmacists interviewed believed that they were very well equipped to do it, and that pharmacists carrying out this work would create a benefit to the patients and would also reduce the GPs’ workload. However, some pharmacists believed that overall responsibility for the patient and the medication prescribed should remain with the GP.

Well I think pharmacists, like I think we’re very well equipped to do it, to, to manage it and particularly in the kind of the new GP contract when there’s going to be a lot more of us about and a lot more of us in GP practices you know I think that would be a good role for us … so however I guess the overall responsibility kind of lies with the, you know I would say the overall responsibility is, it’s the kind of ethos that it lies with the kind of senior whatever person, like it would be the GP really.

PP6, Glasgow pharmacist

GPs who were interviewed stressed their lack of spare time during the working day and an existing heavy workload, meaning that it would not be sustainable for them to review medication to look for anticholinergic medications on top of their existing work. GPs supported the idea of pharmacists carrying out changes in medication for patients, so long as the system worked in such a way that pharmacists doing reviews did not add to the GPs’ workload.

4) Perceived barriers and facilitators to trial participation and the smooth conduct of an ACB reduction trial.

For patients and the public, there were several potential barriers to participating in a trial. Firstly, some expressed the view that they would be wary of a return of symptoms if they changed or stopped medication (Coherence). This was especially true for patients who were taking anti-depressant medication, which takes some time to begin working after starting to take it. Secondly, some patients interviewed were sceptical of the motivations behind switching medications and expressed suspicions that it was a cost-saving exercise rather than a change for their benefit (Coherence).

Not really, I would be prepared if they, if they could give me a good argument for changing then I would definitely do it, if they were to say: “I want to, I want you to try this tablet because I think you’ll have fewer side effects” then I would definitely give it a go yes.

AF15, patient participant at focus group

For HCPs, the main barrier to reducing ACB was insufficient time in a GP appointment to talk to a patient in-depth about medication, especially for patients with multiple long-term conditions. GPs said that it was already difficult in the short time that they had to communicate potential future risks (Collective Action).

The strongest facilitator to participating in a trial for patients/public and HCPs was that there was a desire from both groups to de-prescribe/take fewer tablets (Coherence). The HCPs interviewed agreed that patients were amenable to change if it was explained and discussed with them in detail, and their concerns were heard.

You could try and couch it in quite positive terms, sometimes you do manage to get people off things so don’t want to be too negative about it.

TFGFP18, GP in Glasgow

5) Appraisal Work (Reflexive monitoring): HCP and patient views on the future implementability of this approach to ACB reduction.

Many of the members of the public and patients interviewed stated that, for a pilot study to be successful, they would expect and want to be closely monitored during it and have speedy access to a contact person with whom they could raise any concerns they might have.

You’d like to have somebody to right just say it was you I had your, like, number, and I could phone up and say: “Look it’s (Name) here I’m shaking with this” [overtalk], that’s not for you if you could have a link, a communication link that you could raise any concerns that would be, that would be acted on, you know and you could give you know appropriate guidance in that respect.

P2, Patient participant at focus group

The HCPs interviewed had some suggestions about how ACB could be reduced. They emphasised the need for a usable scoring system, which would be a useful guide to the ACB, and which could be incorporated into existing Information Technology (IT) systems. Any IT system would need to provide information about the ACB score in a useful format; but should avoid causing “pop-up fatigue” i.e., too many irrelevant alerts causing the users to ignore them. HCPs also stressed the importance of long-term follow up, as without it they could not assess if de-prescribing worked.

Some HCPs expressed the view that in some cases, there are no alternatives to an anticholinergic medication which would work as well, for example, one focus group discussed the difficulties with avoiding ACB when prescribing pain-relieving medication in cases where paracetamol does not give sufficient relief from symptoms. GPs also discussed how patients sometimes had the expectation that there should be “a pill for every ill,” making it difficult to get the balance right. HCPs also spoke about previous experiences of de-prescribing initiatives which did not succeed or which increased their workload (Reflexive Monitoring).

6) Appraisal Work (Reflexive monitoring). Public and patients’ willingness to be contacted for participation in a future trial of this kind and general views about a trial.

Most members of the public and patients interviewed said that they were willing to be contacted about future participation in a trial and were generally positive about the idea of examining prescribing in this way.

The findings are summarised in Table 2.

**TABLE 2:** Summary of findings from interviews and focus groups.

**1) Sense-making (coherence)**	
Patient and professional views of a trial of de-prescribing/switching	
Patients	• Learning about their conditions and medications
	• Sourcing information from HCPs and the internet
	• Fear of learning too much
	• Generally positive about running de-prescribing/switching trial
HCPs	• Varied knowledge of ACB depending on role
	• GPs wary of adding to already heavy workload
	• General positive attitude towards de-prescribing/switching
**2) Relationship work (cognitive participation)**	
Patient and professional views on how to best communicate information about such a trial	
Patients	• HCPs need to take more time to explain medications and side-effects
HCPs	—
**3) Enacting work (collective Action)**	
Patient and professional views regarding who should undertake such medication changes, for example, pharmacists or GPs?	
Patients	• Intervention would mean that they would need to take a more active role in their healthcare
	• Patients who had experienced prescribing pharmacists were happy for pharmacists to prescribe
HCPs	• Would need more time with patient appointments to discuss the new way of working
	• Change in roles required i.e. widen prescriber role
	• Pharmacists to prescribe but overall responsibility to remain with GP
	• GPs wary of adding to already heavy workload
**4) Barriers and facilitators**	
Perceived barriers and facilitators to trial participation and the smooth conduct of an ACB reduction trial	
Barriers	
Patients	• Wary of symptoms returning (especially depression)
	• Sceptical of motivations. Suspicious it’s cost-saving
HCPs	• Insufficient time in patient appointments for discussion
Facilitators	
Patients	• Desire to take fewer tablets
HCPs	• Desire to de-prescribe
	• Patients amenable to change if sufficient time take to explain to them and hear their concerns
**5) Appraisal work (reflexive monitoring)**	
HCP and patient views on the future implementability of this approach to ACB reduction	
Patients	• Wanted trial participants closely monitored
HCPs	—
**6) Appraisal work (reflexive monitoring)**	
Public and patients’ willingness to be contacted for participation in a future trial of this kind and general views about a trial	
Patients	• Public and patients’ were willing to be contacted about participation in a future trial of this kind and made suggestions about how best to recruit participants for such a trial
HCPs	—

## Discussion

Our study highlights that members of the public, patients and HCPs are all open to the idea of ACB reduction through interventions that promote de-prescribing or medication changes. It was clear that patients and members of the public were eager to better understand what different medications were for and why they should be taking them. Poor understanding of treatments is a longstanding problem in healthcare (Browne et al., 2014). However, while the key stakeholders were supportive of the idea of the future trial there were several caveats and recommendations. The participating members of the public and patients were clear that PPI would be essential from the outset. They thought that it would be essential to ensure patient engagement from the beginning to enable concerns and potential pitfalls of anticholinergic de-prescribing to be addressed from the outset. They emphasised the importance of clear communication so that patients involved in any trial have a very clear understanding of the rationale and aims of a trial to minimise the potential for misconceptions about the reasons for ACB reduction. They also suggested it would be important to provide access to a point of contact for patients throughout the life of a trial to address queries or concerns. While HCPs were generally positive about the idea of de-prescribing or medication switching it was clear that taking steps to minimise the workload implications of any ACB reduction trial would be important. GPs emphasised that their workloads were heavy and time constraints would pose a major barrier to the potential success of any de-prescribing intervention. GPs suggested that more effective utilisation of Information Technology (IT) systems might make the work of de-prescribing easier. There was a consensus that pharmacists are best placed to carry out ACB reviews, though overall responsibility for patient medication should remain with the GP.

While there is a broad range of literature addressing barriers and facilitators to de-prescribing (Anderson et al., 2014; Luymes et al., 2016, Reeve et al., 2013) there is little focused specifically on de-prescribing of anticholinergic medication (Kouladjian et al., 2016; Gnjidic et al., 2010). The existing qualitative work exploring barriers and facilitators to ACB reduction have only involved HCPs (Kouladjian et al., 2016; Gnjidic et al., 2010) and to the best of our knowledge, there have been no studies involving patients or members of the public that have explored the issue of anticholinergic de-prescribing. The work with HCPs did show, as in our study, that good communication with patients and other HCPs would be important and that assigning responsibility for prescribing decisions to one named individual would be helpful (Kouladjian et al., 2016; Gnjidic et al., 2010). This work showed that resource constraints were also an issue, and the wider de-prescribing literature confirms time constraints can be a barrier to de-prescribing (Anderson et al., 2014). Our work resonates with the wider de-prescribing literature where patients have been involved (Steinman and Reeve, 2020) which emphasises the importance of engaging with patients in the de-prescribing process, ensuring they understand “why” a medication should be switched or stopped. Our findings that patients were often unclear of the purpose of different medications resonates with an extensive literature that highlights how patients and caregivers often have inadequate understanding of medications, their side effects, and limitations (Browne et al., 2014) and indeed often turn to the internet to address information gaps (Fox and Duggan, 2013). Our work is novel in that it explicitly sought views on the design of a proposed ACB reduction trial but suggests any intervention will be more likely to be successful if patients and their supporters better understand the reasons for treatments. Thus, any future trial will need to help people better understand the pros and cons of different medications. Making such information easily comprehensible will be an important issue for any future de-prescribing interventions.

## Study Limitations/Strengths

Our study has certain limitations. The study was conducted in Scotland, which provides universal access to healthcare, and thus these qualitative findings may not be generalisable to other healthcare contexts. While we did include non-English speakers, most of our sample were Caucasian, so it will be important to conduct future work with other ethnic groups. A clear strength of the study is the involvement of members of the public, patients, and HCPs as well as our use of a robust theoretical framework, NPT, to help us conceptualise our data.

## Clinical Implications

While our findings will primarily be used to feed into the design of a proposed ACB reduction trial. Our study suggests that members of the public, patients and HCPs are all open to the idea of ACB de-prescribing. Members of the public and patients were clear that they would like to increase their understanding of the medications that they are prescribed and that they would be open to de-prescribing if it was viewed as helpful to their wellbeing. Good communication is definitely a key issue to underpin de-prescribing or medication switching initiatives. However, time constraints are a major barrier to de-prescribing, and therefore if clinicians and policy makers wish to promote de-prescribing then structural changes to the way we deliver health care services, whether through enhanced use of IT or other mechanisms will be required to enable HCPs, to have the capacity to undertake such work.

## Data Availability

Data are available upon request to the corresponding author. As data contain information which may lead to the identification of study participants, funding would be required to support anonymisation of the data.

## Supplementary Material

Supplementary Table S1 This is a table which explains how the study meets the “Standards for Reporting Qualitative Research (SRQR)”.

Data Sheet 1 Flyer used to advertise focus groups. This flyer also lists commonly prescribed medications with anticholinergic properties.

Data Sheet 2 Topic guide for patients. This is the topic guide used when interviewing patients and holding focus groups with patients and members of the public.
