# Supplementary material for: Understanding Stakeholder Views Regarding the Design of an Intervention Trial to Reduce Anticholinergic Burden: A Qualitative Study
Source: Front Pharmacol. 2021 Nov 12;12:608208. doi: 10.3389/fphar.2021.608208 (PMC8633300; doi:10.3389/fphar.2021.608208)
Supplement: Supplementary file 2 [file DataSheet1.PDF]

We're looking for discussion group participants!

TOPIC: Reducing/changing certain medications which can have side effects.

WHO? We're looking for older adults (age 65+) who take two or more of the medications listed overleaf to share their experiences of taking the medications and their views on stopping or changing them.

WHAT? Participants are asked to spend an hour and a half in a discussion group about the topic. Travel expenses will be paid.

WHY DO IT? By taking part, you can help to benefit people using such medicines in future. You will also receive a £50 gift voucher as a thank you.

Want to know more?
Then ring Yvonne Cunningham at:
0141 330 2041 or
email: yvonne.cunningham@glasgow.ac.uk

The research is being carried out by researchers in the Institute of Health & Wellbeing at the University of Glasgow and is funded by the Chief Scientists Office.

University of Glasgow

VIA VERITAS VITA

ALLIANCE
HEALTH AND SOCIAL CARE
ALLIANCE SCOTLAND
people at the centre

If you are aged over 65 and are taking two or more of any of the following medicines, then we would like to talk to you! Please contact Karen Wood at 0141 3302816 or karen.wood@glasgow.ac.uk

Antihistamines
Allegra
Allercalm
Allerief
Atarax
Avomine
Benadryl Allergy
Cetirizine
Chlorphenamine
Clarityn
Fexofenadine
Hayleve
Hydroxyzine
Loratadine
Phenergan
Piriteze
Piriton
Promethazine
Sominex
Telfast
Zirtek

Diuretic/Water Tablets
Diumide-K Continus
Frumil
Frusene
Furosemide
Lasilactone
Lasix

Stomach or Indigestion medications
Cimetidine
Ranitidine
Tagamet
Zantac
Zantac 75
Zantac 75 Relief

Bladder medications
Cystrin
Detrusitol
Detrusitol
Ditropan
Kentera
Lyrinel XL
Oxybutynin
Solifenacin
Tolterodine
Vesicare

Pain Relief medications
Amitriptyline
Carbagen
Carbamazepine (also for epilepsy)
Co-codamol
Codeine
Co-dydramol
Desyrel
Dihydrocodeine
Elavil
Endep
Kapake
Molipaxin
Paramol
Remedeine
Solpadol
Tegretol
Tegretol XR
Trazodone
Triptafen
Trittico
Tylex
Vanatrip

Blood Thinners/Anti-coagulants
Warfarin

Irritable bowel /Anti-diarrhoeal/ Motion sickness medications/Labyrinthitis/Nausea /Vomiting
Bentyl
Buscopan
Dicyclomine
Dicycloverine
Dioraleze
Hyoscine
Imodium
Loperamide
Merbentyl
Pro-Banthine
Propantheline
Scopolamine

Anti-depressant medications
Cipralex
Cipramil
Citalopram
Escitalopram
Fluoxetine
Lustral
Mirtazapine
Oxactin
Paroxetine
Prozac
Seroxat
Sertraline
Zispin SolTab
