# Supplementary material for: Understanding Stakeholder Views Regarding the Design of an Intervention Trial to Reduce Anticholinergic Burden: A Qualitative Study
Source: Front Pharmacol. 2021 Nov 12;12:608208. doi: 10.3389/fphar.2021.608208 (PMC8633300; doi:10.3389/fphar.2021.608208)
Supplement: Supplementary file 3 [file DataSheet2.docx]

##
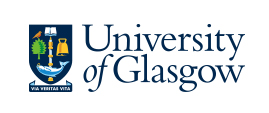

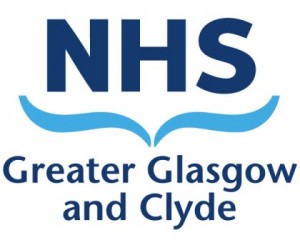

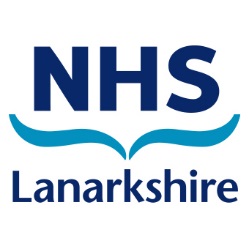


##
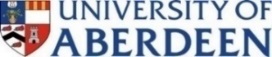


***Perspectives of health professionals and patients on reducing anticholinergic burden by stopping or switching medications: A qualitative study***

Topic guides for patients

Provide consent form for participants to read and sign (ask if they have any queries before they sign)

START WITH INTRODUCTIONS - My name is _______ and I am a researcher at the University of Glasgow. Thank you for agreeing to participate in this interview.

- Just to remind you that this study is to help us understand your experiences with your medication and also your opinion regarding reducing anticholinergic side effects by stopping and/or switching medication.

- When I say anticholinergic side effects I mean these side effects are palpitations, dizziness, drowsiness, blurred vision, increased heart rate, confusion, dry mouth and eyes and urinary retention. These types of side effects are often occur due to medicines that are prescribed for high blood pressure, heart disease and lung disease.

- We would like to know your views about reducing these side effects by switching a medication with another one or stopping these medication.

-You must receive Information Leaflet by post. Have you got any questions?

- I am not a clinician, I am a researcher so I am not in a position to make any judgement about what you say. I am really just interested in your views and experiences. Your doctors will not know about what you said and also your discussion will have no effect on your treatment or care.

- I am going to ask you a number of questions today. If any questions are not clear please let me know. All information collected will be strictly confidential. I would like to record our chat today so that we have an accurate record of it and I don’t have to rely on my memory. Are you happy for the interview to be recorded? Any questions before we start the chat?

| **Topic** | **Questions** | **Prompts/Probes** |
| --- | --- | --- |
| Background | To start with I will ask you a few questions about your background?   1. What type of health issues are you currently being treated for? 2. What medicines are you taking now? 3. How long have you been taking these medicines for? | -If you don’t remember, don’t worry, that’s ok. |
| Experience of medication | 1. Can you tell me a bit about your experiences with your medicines? | - Are you feeling any better than before? - Anything not very pleasant? - Anything else? |
| unexpected or unwanted effects | 1. Have you ever experienced of any unexpected or unwanted effects? | If yes   - What are they? - How did you deal with these side-effects? - Did you seek any help? From whom? - Was it helpful/reduced the side effects?   If No   - Remind these: As mentioned earlier about various side effects of medication like palpitations, dizziness, drowsiness, blurred vision, increased heart rate, confusion, dry mouth and eyes and urinary retention. - Have you experienced any of these? |
| Information about side effects | 1. How much information did you get from your GPs, specialists or health care professionals tell you about anticholinergic side effect? | If yes   - What information did you get? - Did they discuss any alternatives/options to avoid these effects? - How did the discussion go?/ What did they tell you? |
| Concerning about medications | 1. You might have answered these already, but can I ask again   Have you got any other concerns with your own medication at the moment? | If yes   - Did you discuss these further with anybody? - How did you manage with side effects of your medication? - Have you ever discuss this with health professionals (like doctors, pharmacists, nurses)?   If no, Ok thank you. |
| Available information to manage side effects | 1. Is there any information/support out there to manage these side effects? | If yes   - What is information? - How did you get them? from whom? - How useful did you find them?   Is there anything else that you think would have been helpful?  If no   - Any suggestions regarding what would have been helpful to manage these issues? - -How NHS could have helped you? |
| Person to manage side effects | 1. Who do you think is the most important person (who could guide you) to manage your side effects? | Why?  -Anyone else? |
| Stopping/Changing medications | 1. Would you be willing to stop or change medication to one that have less side effect if your prescribers or pharmacists suggest?   Remind again- I am not a clinician, I am a researcher so I am not in a position to make any judgement about what you say. We are only interested in your views. | If yes   - What is the reason that you are willing to stop or change?   If no   - What is the reason that you are not willing to stop or change? |
| Future research study | 1. Would you be willing to take part in a research study of medication switching or stopping (e.g. some people have medication stopped or altered and others do not)? | If yes   - Why?   If no   - Why? - What do you think might stop you or others taking part in such a research study?   I understand that you do not want to take part but any suggestions for us such as   - What would be the best way of recruiting patients for this type of research study? - For example (if they don’t talk) (face-to-face by a health professional or letters by post etc.)? Anything else? |
|  | 1. Although there is no obligation, would you like to be contacted for any future study of this kind? | If yes   - What is the best way to contact you? By post/telephone? |

That’s all the questions I have for you- Is there anything else that you feel important and we have not discussed today?

**- Is that okay if I contact you if there are any points of clarification?**

***Thank you very much again for your time taking part in this study.***

***-SWITCH-OFF***
